# Shuttle-like supramolecular nanostructures formed by self-assembly of a porphyrin via an oil/water system

**DOI:** 10.1186/1556-276X-6-529

**Published:** 2011-09-23

**Authors:** Peipei Guo, Penglei Chen, Minghua Liu

**Affiliations:** 1 Beijing National Laboratory for Molecular Science, CAS Key Laboratory of Colloid, Interface and Chemical Thermodynamics, Institute of Chemistry, Chinese Academy of Sciences, No. 2 Zhongguancun Beiyijie, Beijing, People's Republic of China

## Abstract

In this paper, in terms of the concentration of an aqueous solution of a surfactant, we investigate the self-assembly behavior of a porphyrin, 5, 10, 15, 20-tetra(4-pyridyl)-21*H*, 23*H*-porphine [H2TPyP], by using an oil/water system as the medium. We find that when a chloroform solution of H2TPyP is dropwise added into an aqueous solution of cetyltrimethylammonium bromide [CTAB] with a lower concentration, a large amount of irregular nanoarchitectures, together with a small amount of well-defined shuttle-like nanostructures, hollow nanospheres, and nanotubes, could be produced. While a moderate amount of shuttle-like nanostructures accompanied by a few irregular nanoarchitectures, solid nanospheres, and nanorods are produced when a CTAB aqueous solution in moderate concentration is employed, in contrast, a great quantity of shuttle-like nanostructures together with a negligible amount of solid nanospheres, nanofibers, and irregular nanostructures are manufactured when a high-concentration CTAB aqueous solution is involved. An explanation on the basis of the molecular geometry of H2TPyP and in terms of the intermolecular π-π interactions between H2TPyP units, and hydrophobic interactions between CTAB and H2TPyP has been proposed. The investigation gives deep insights into the self-assembly behavior of porphyrins in an oil/water system and provides important clues concerning the design of appropriate porphyrins when related subjects are addressed. Our investigation suggests that an oil/aqueous system might be an efficient medium for producing unique organic-based nanostructures.

## Introduction

Currently, tremendous efforts have been devoted to open up various facile methodologies for the fabrication of nanostructured materials with novel morphologies since the fascinating architectures and the unique physicochemical properties of the produced nanomaterials make them of considerable interest as promising components for chemical/biochemical sensors, opto- and nano-electronic devices, bionanotechnology, and so forth [1-14]. Among various nanostructured materials, those with well-defined discrete architectures, typically exemplified by nanofibers, nanotubes, nanorods, nanospheres, nanocubes, etc., have so far been intensively investigated [1-14]. Besides these sophisticated typical nanomaterials, those with complicated yet well-defined morphologies, for example, cauliflower-like nanostructures, nanopolyhedrons, nanotetrapods, nanosprings, nanospirals, nanospindles, nanoshuttles, etc. [15-30], have also attracted much attention owing to their extraordinary physicochemical properties. With respect to this issue, a paramount of inorganic-based systems have been developed [1-6,15-24], while examples in terms of organic-based systems are relatively fewer [4-6,10,11]. As a matter of fact, in contrast to inorganic nanomaterials [1-6], organic nanostructures have peculiar electronic and optical properties and can render impressive varieties and flexibilities in molecular design and tunability of physicochemical properties [7-14]. This makes the organic-based nanostructures promising candidates for nanoscience and nanotechnology. Consequently, an exploration on the fabrication of organic nanostructures with unique yet well-defined morphologies should be an important issue to be explored intensively.

When considering the fabrication of organic-based nanostructured materials, supramolecular assembly, which aims at manufacturing sophisticated, organized molecular associations through various non-covalent interactions, including π-π interactions, hydrophobic interactions, electrostatic interactions, etc., has currently been demonstrated to be an excellent strategy. It provides fertile, new, and promising opportunities for nanoscience and nanotechnology, material science, biological science, and so on [7-14,31]. Various fascinating organic-based nanomaterials have so far been manufactured in terms of diverse solution-based self-assembly technique, template-induced self-assembly process, physical vapor deposition, etc. [7-14,25-30], where the surfactant-assisted self-assembly [SASA] has been considered to be an important solution-based method owing to its nice adaptability, simplicity, and reproducibility [32,33].

Among various building blocks for the fabrication of supramolecular nanostructures, porphyrins have been reported to be one of the most useful units. This is promoted by their multifunctionality, biocompatibility, flexible and tunable molecular structure by chemical modification, rigid and planar molecular skeleton, which make porphyrin-involved nanomaterials have rich assembly, physicochemical and biochemical properties [26,27,29,32-39]. For example, we have recently demonstrated that a metal porphyrin, zinc 5, 10, 15, 20-tetra(4-pyridyl)-21*H*, 23*H*-porphine [ZnTPyP], could be organized to form various well-defined nanostructures in a controllable manner via a SASA method through an oil/water system [39]. In that case, we showed that depending on the aging time and the concentration of the aqueous solution of the surfactant, cetyltrimethylammonium bromide [CTAB], hollow or solid nanospheres, nanotubes, nanorods, and nanofibers could be facilely obtained. Therein, we suggested that the intermolecular π-π interactions between ZnTPyP units together with the hydrophobic interactions between ZnTPyP and CTAB played an important role during the assembly process.

**Figure 1:**
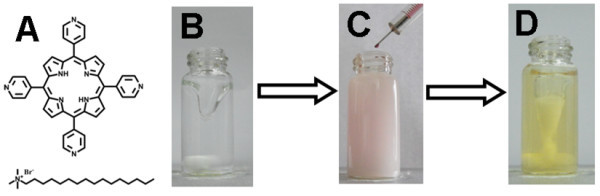
**Fabrication of H2TPyP-based nanostructures via an oil/water medium**. (**A**) Molecular structures of 5, 10, 15, 20-tetra(4-pyridyl)-21*H*, 23*H*-porphine (H2TPyP) and cetyltrimethylammonium bromide (CTAB). (**B**), (**C**) Self-assembly process via an oil/water medium, where (**B**) shows the original transparent CTAB aqueous solution and (**C**) indicates the addition of a H2TPyP chloroform solution to a CTAB aqueous solution. (**D**) Solution obtained after stirring is maintained for 15 min.

Actually, the following issue should generally be taken into account to obtain well-defined organic-based nanostructures through self-assembly [8,14]: (1) internal factors, namely, tuning the molecular arrangements in the nanostructures by modifying the molecular structures of the involved building blocks, and (2) external factors, that is, optimizing self-assembly conditions such as the temperature, concentration, etc. From this point of view, an investigation on the assembly behavior of 5, 10, 15, 20-tetra(4-pyridyl)-21*H*, 23*H*-porphine [H2TPyP] (Figure 1), a free-base structurally allied counterpart of ZnTPyP, might be able to provide us deeper insights into the SASA process occurring in the oil/water system.

On the basis of the aforementioned brief review, we herein investigate the assembly behavior of H2TPyP via a process similar to that for ZnTPyP [39]. We find that a large amount of irregular nanoarchitectures, together with a small amount of shuttle-like nanostructures, hollow nanospheres, and nanotubes, could be produced using a lower concentration of a CTAB aqueous solution. When a CTAB aqueous solution with a moderate concentration is used, a moderate amount of shuttle-like nanostructures accompanied by a few irregular nanoarchitectures, solid nanospheres, and nanorods could be produced, while a good quantity of shuttle-like nanostructures, together with a very small amount of solid nanospheres, nanofibers, and a negligible amount of irregular nanostructures could be predominantly manufactured when a high-concentration CTAB aqueous solution is employed. An explanation on the basis of the molecular geometry of H2TPyP and in terms of the intermolecular π-π interactions between H2TPyP molecules and the hydrophobic interactions between CTAB and H2TPyP has been proposed. Although various porphyrin-involved organic nanostructures [26,27,29,32-39] and inorganic shuttle-like nanostructures have been previously produced [22-24], this might be the first example showing that porphyrin-based shuttle-like nanoarchitectures could be facilely produced via an oil/water medium. The study gives deep insights into the self-assembly behavior of porphyrins in an oil/water system and provides important clues to the designing of appropriate porphyrins when related subjects are addressed.

## Experimental

### Chemicals and reagents

CTAB (99%, Alfa Aesar) and H2TPyP (97%, Aldrich) were used as received without further purification. Milli-Q water (18 MΩ cm) and distilled chloroform were used as the solvents for CTAB and H2TPyP, respectively.

### Methods and procedures

The process for the synthesis of H2TPyP-based nanostructures via the oil/water medium is similar to those described for ZnTPyP [39]. Typically, 400 μL of a chloroform solution of H2TPyP (2 × 10^-4 ^M) was added dropwise into a 10-mL aqueous solution of CTAB under vigorous magnetic stirring within 1 to 2 min. The concentrations of the CTAB aqueous solutions were 0.225, 0.9, and 4.5 mM for the samples named as I, II, and III, respectively. Soon after adding H2TPyP chloroform solution, an opaque solution was obtained; a transparent yellowish solution was obtained after vigorous stirring was maintained for 15 min, as shown in Figure 1. The solution was then kept at room temperature without disturbing for a desired time for aging, after which the UV/vis spectra of the solutions were measured. The formed nanostructures were dramatically washed with Milli-Q water several times via repeating filtration or centrifugation, after which they were subjected to various characterizations. The Millipore filter (Whatman) used for the filtration has a pore size of 200 nm. In the case of centrifugation, a rotation speed of 10, 000 rpm is adopted. We found that similar results could be obtained for the samples washed by these two methods. Subsequently, the nanostructures were characterized by low-resolution transmission electron microscopy [LRTEM], high-resolution transmission electron microscopy [HRTEM], fast Fourier transformation [FFT], energy-dispersive X-ray spectroscopy [EDX], and scanning electron microscopy [SEM].

### Apparatus

A JASCO UV-550 spectropolarimeter was used for the UV/vis spectral investigation. The SEM measurements were performed using a Hitachi S-4800 system. The LRTEM and HRTEM images of the nanostructures were obtained with a FEI Tecnai G^2 ^F20 U-TWIN, where the accelerating voltages were set as 200 and 80 kV, respectively. Elemental analysis was achieved using energy-dispersive X-ray spectroscopy on the FEI Tecnai G^2 ^F20 U-TWIN.

## Results and discussions

### SEM images of H2TPyP-involved nanostructures

**Figure 2:**
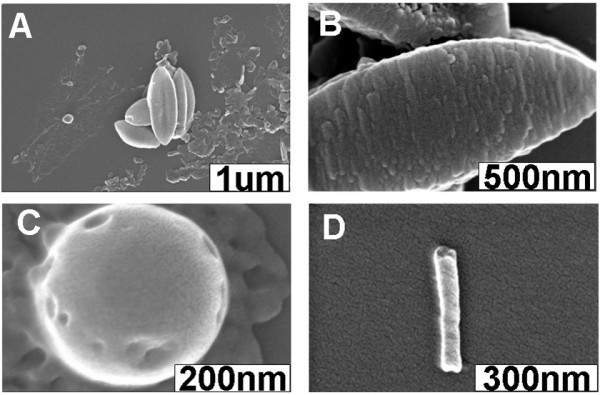
**SEM images of various nanostructures obtained from sample I**. (**A**) SEM image of the irregular nanoarchitectures accompanied by several shuttle-like nanostructures. (**B**) High-resolution SEM image of the shuttle-like nanostructures. (**C**) SEM image of a hollow nanosphere. (**D**) SEM image of a nanotube. The aging time is 15 min.

Experimentally, the concentrations of the CTAB aqueous solutions were 0.225, 0.9, and 4.5 mM for the samples named as I, II, and III, respectively. As shown in Figure 2, large amounts of irregular nanostructures (above 70%), together with some interesting discrete shuttle-like architectures (about 20%) with a dimension of approximately 150 to 1, 000 nm, hollow nanospheres, and nanotubes, are observed from the SEM of sample I, when the aging time is 15 min. Moreover, it is found that the samples obtained using a longer aging time, that is 3 h or 3 days, display similar results (figures not shown), suggesting that the aging time could hardly affect the assembly behavior of the H2TPyP molecules in the present case. As we have found previously [39], when the aging time is extended from 15 min to 3 days, well-defined hollow nanospheres and nanotubes could be obtained in sequence from the corresponding sample I formulated by ZnTPyP units. The present results suggest that our H2TPyP could display an essentially different assembly behavior from that of ZnTPyP, although some hollow nanospheres and nanotubes could also be obtained from the present system.

**Figure 3:**
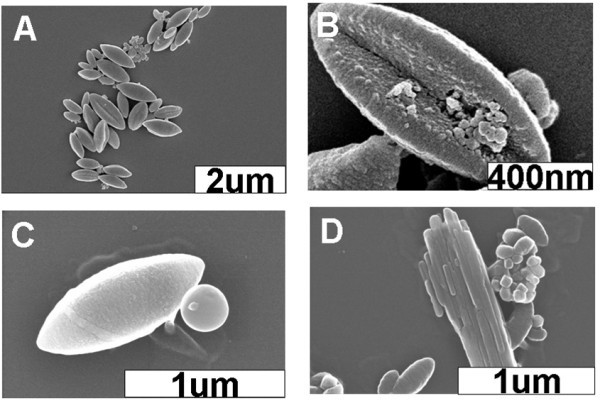
**SEM images of various nanostructures obtained from sample II**. (**A**) SEM image of the shuttle-like nanostructures accompanied by several irregular nanoarchitectures. (**B**) High-resolution SEM image of the shuttle-like nanostructures together with some irregular nanoarchitectures. (**C**) SEM image of a solid nanosphere and a shuttle-like nanostructure. (**D**) SEM image of several nanorods, nanoshuttles, and irregular species. The aging time is 15 min.

In contrast, in the cases of sample II, more than 65% of well-defined shuttle-like nanoarchitectures with a dimension of approximately 150 to 1, 000 nm, accompanied by approximately 30% of irregular nanostructues and approximately 2% of solid nanospheres and nanorods, respectively, could be statistically observed when the sample is aged for 15 min, as presented in Figure 3. Similarly, as what we have found from sample I, the aging time could not affect the morphology of the formulated nanostructures of this system. As a matter of fact, for the corresponding sample II in the case of ZnTPyP, well-defined solid nanospheres and nanorods could be formulated, respectively, when aging times of 15 min and 3 days are employed [39]. These results suggest that H2TPyP and ZnTPyP indeed have different assembly behaviors in our oil/water system, although there are only small differences in their molecular structure.

**Figure 4:**
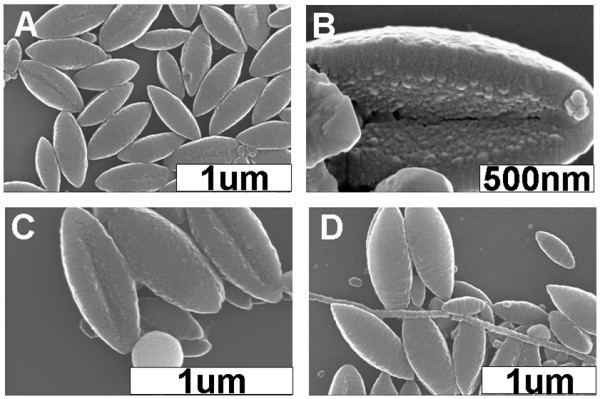
**SEM images of various nanostructures obtained from sample III**. (**A**) SEM image of the shuttle-like nanostructures. (**B**) High-resolution SEM image of a shuttle-like nanostructure. (**C**) SEM image of a solid nanosphere and several shuttle-like nanostructures. (**D**) SEM image of several nanoshuttles, nanofibers, and irregular species. The aging time is 15 min.

When comparing samples I and II, it could be seen that the relative content of the irregular and shuttle-like nanostructures decreases and increases, respectively, when the concentration of the CTAB aqueous solution increases from 0.225 to 0.9 mM. This suggests that a higher CTAB concentration might favor the formation of unique shuttle-like nanoarchitectures. Along this line, we investigated the self-assembly behavior of H2TPyP using an even higher concentration of CTAB aqueous solution. As shown in Figure 4, the statistical results obtained from sample III suggest that nearly more than 95% of the shuttle-like nanostructures, accompanied by a small percentage of solid nanospheres and nanofibers, and a relatively negligible percentage of irregular nanostructures, could be obtained for the samples with aging times of 15 min, 3 h, and 3 days. These results confirm that the interesting shuttle-like nanomaterials could be predominately formulated using a CTAB solution with a higher concentration. Comparatively, for the corresponding sample III of ZnTPyP, well-defined solid nanospheres and nanofibers could be obtained, further confirming the different SASA behavior of these two molecules.

**Figure 5:**
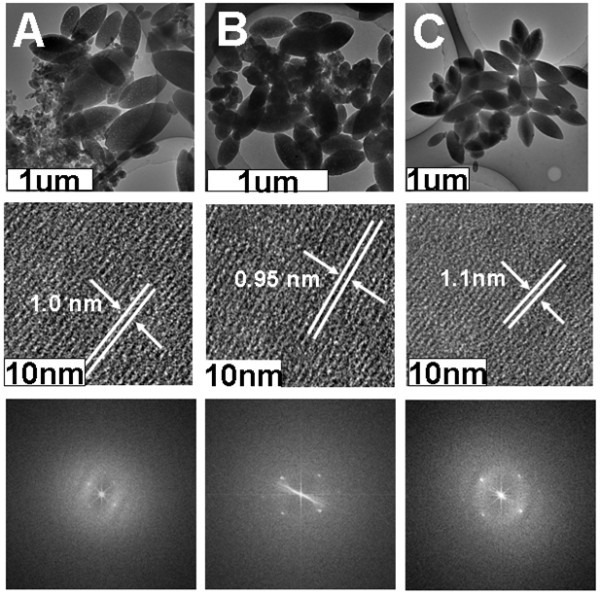
**TEM and FFT images of the shuttle-like nanostructures**. Images obtained from samples I (**A**), II (**B**), and III (**C**). *Top panel*: LRTEM images of the shuttle-like nanostructures obtained from sample I (**A**), II (**B**), and III (**C**). *Middle panel*: corresponding HRTEM images of the shuttle-like nanostructures shown in the top panel. *Bottom panel*: corresponding FFT of the HRTEM images shown in the middle panel. The aging time for all the samples is 3 h.

The morphology of our shuttle-like nanostructures was also investigated by LRTEM. As shown in the top panels of Figure 5, for all the samples, shuttle-like nanoarchitectures, whose basic morphology and dimension are similar to those detected by SEM, could be distinctly observed. This suggests that well-defined, discrete shuttle-like H2TPyP-involved nanostructures could indeed be formulated via our SASA protocol.

### UV/vis spectra of H2TPyP-involved nanostructures

**Figure 6:**
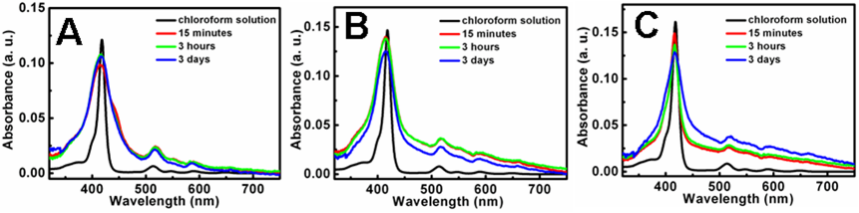
**UV/vis spectra**. Spectra of samples I (**A**), II (**B**), and III (**C**) as a function of the aging time.

As known, porphyrin species could typically form J- or H-type aggregates when they are organized to form supramolecular assemblies [40-45]. Their UV/vis spectra are widely acknowledged to be a powerful method that could provide deep insights into their assembly behavior owing to the well-understood diagnostic and intensive Soret band around 420 nm [39-45]. Generally, on the basis of the exciton coupling model proposed by Kasha and coworkers, for characteristic J-type aggregates, the Soret band would split into two bands, which manifest themselves as a narrow and bathochromic-shifted band, and a broadened and hypsochromic-shifted band as well. The former and latter bands are ascribed to the transition moments parallel and perpendicular to the aggregate axis, respectively. For the typical H-aggregates, a broadened and blue-shifted Soret band could be observed [40-45]. The UV/vis spectra of our nanostructures as a function of the aging time were measured, as shown in Figure 6. For H2TPyP dispersed in chloroform solution, it can be seen that its UV/vis spectra display a sharp Soret band at 418 nm and four Q-bands around 513, 546, 588, and 648 nm. In the case of sample I aged for 15 min, the Soret band becomes broadened and blue shifted to 416 nm, as presented in Figure 6A. No distinct Soret band corresponding to the H2TPyP monomer could be observed and the UV/vis spectra display no aging time dependence, as suggested by the nearly unchanged spectral curves displayed by the sample aged for 3 h and 3 days. In the cases of samples II and III where CTAB aqueous solutions with higher concentrations are employed, approximately similar results were observed, as shown in Figures 6B and 6C, respectively. These results suggest that our H2TPyP molecules form H-type supramolecular aggregates through intermolecular π-π interactions [40-45] upon the addition of a chloroform solution into the CTAB aqueous solution. Accompanied by information obtained from the SEM images, where the morphology of the formulated nanostructures exhibit no aging time dependence, the present UV/vis spectra indicate that the observed H2TPyP-based nanostructures might be suddenly formed soon after the chloroform solution of H2TPyP is added into the aqueous solution of CTAB.

### TEM images of H2TPyP-involved nanostructures

Moreover, in order to reveal the internal structure of our shuttle-like nanoarchitectures, their HRTEM and FFT images were investigated, respectively. During the experiments, we found that our organic nanostructures suffer a fast amorphization under the strong electron beam (with an accelerating voltage of 200 kV) illumination. Therefore, the HRTEM was carried out with an accelerating voltage of 80 kV, where it was find that amorphization could be decelerated to some extent. As shown in the middle and bottom panels of Figure 5, distinct lattice fringes could be detected from all of the formulated shuttle-like nanoarchitectures. An interlattic spacing of about 1 nm could be obtained, as indicated by the FFT analysis. This value is very close to the side length of H2TPyP square, which was calculated to be approximately 1.1 nm. Accompanied by information deduced from the UV/vis spectra, these results suggest that our shuttle-like nanostructures are essentially composed of H-type molecule associations.

### An explanation for the formation of the shuttle-like supramolecular nanostructures

It has been demonstrated that the formation of a porphyrin/surfactant complex is facilitated primarily by the electrostatic or hydrophobic interactions between these two components [46-49]. In the case of ionic porphyrin/nonionic surfactant or nonionic porphyrin/ionic surfactant systems, the hydrophobic interactions between the alkyl chain of the surfactant and the π system of porphyrin play an essential role [32,46,47]. In our present case, the employed porphyrin is a nonionic compound. Consequently, the electrostatic interactions between porphyrin and the surfactant are negligible. Thus, we could simply propose that three kinds of intermolecular interactions, i.e., π-π interactions between porphyrin molecules (H2TPyP/H2TPyP), hydrophobic interactions between porphyrin and CTAB molecules (H2TPyP/CTAB), and CTAB/CTAB interactions, compete with each other during the SASA process.

As has been demonstrated, for zinc porphyrins, owing to the large size of the zinc cation, Zn generally stands out from the mean macrocyclic plane of porphyrin, and instead of a planar geometry, zinc porphyrin always exhibits a bent molecular geometry [50,51]. This could cause a somewhat steric hindrance between zinc porphyrin molecules when they are assembled through intermolecular π-π interactions [51]. Previously, our experimental facts suggested that the ZnTPyP molecule formed a ZnTPyP/CTAB complex with CTAB through the intermolecular hydrophobic interactions between the alkyl chain of CTAB and the π system of ZnTPyP upon coming into contact with CTAB [39]. This was a result of the dynamic competition between the aforementioned three types of intermolecular interactions. After this, owing to their thermodynamic superiority, ZnTPyP units formed assemblies via π-π interactions gradually during the aging, resulting in the formation of various well-defined nanostructures [39].

**Figure 7:**
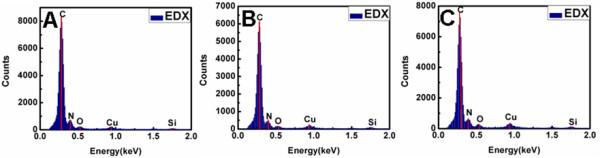
**Energy-dispersive X-ray spectroscopy**. EDX of the shuttle-like nanostructures obtained from samples I (**A**), II (**B**), and III (**C**). The aging time for all the samples is 3 h. EDX, energy-dispersive X-ray spectroscopy.

In contrast, in the present case of free-base porphyrin H2TPyP, there is no big zinc cation in its central cavity and it has a nice planar configuration [52,53]. Accordingly, compared with its zinc counterpart, there exists a relatively smaller intermolecular steric hindrance between H2TPyP molecules, resulting in more favorable intermolecular π-π interactions. As a result of both dynamic and thermodynamic preference, most of the H2TPyP molecules could thus directly form aggregates suddenly via intermolecular π-π interactions without the formation of a H2TPyP/CTAB complex. This could be confirmed by the EDX of the formed nanostructures shown in Figure 7, where it could be seen that almost no signal from bromine could be detected. Another evidence supporting this proposal is that yellowish precipitates could be easily observed from the bottom of the flask when the aqueous dispersions of our H2TPyP-based nanostructures are standing for several hours. In contrast, in the case of ZnTPyP-based nanostructures, distinct signals originated from bromine could be detected by means of EDX, and the dispersion could keep its transparency with negligible precipitates for several months [39]. Thus, the present result suggests the poor dispersibility of H2TPyP-based nanostructures in an aqueous system owing to the lack of assistance from CTAB surfactants.

At the same time, it should also be pointed out that, still, a small amount of H2TPyP molecules could act as ZnTPyP during the SASA since small amounts of hollow nanospheres and nanotubes, solid nanospheres and nanorods, and solid nanospheres and nanofibers, which are predominately formed in the corresponding samples of ZnTPyP systems, could after all be observed from samples I, II, and III of H2TPyP, respectively.

**Figure 8:**
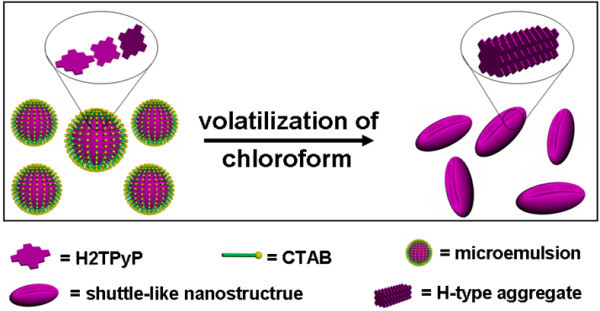
**Formation of shuttle-like H2TPyP nanostructures**. Possible explanation proposed for the formation of shuttle-like H2TPyP nanostructures via an oil/water medium. CTAB, cetyltrimethylammonium bromide; H2TPyP, 5, 10, 15, 20-tetra(4-pyridyl)-21*H*, 23*H*-porphine.

On the basis of the aforementioned experimental facts and analyses, we now could propose a possible explanation for the interesting phenomena observed from the H2TPyP system, as schematically shown in Figure 8. When H2TPyP molecules in chloroform solution were dropwise added into the CATB aqueous solution with a high concentration, the oil phase could be spherically dispersed in the aqueous phase with the assistance of abundant surfactant, resulting in an opaque microemulsion system due to the incompatibility of chloroform and water [52-54]. In most of the spherical emulsions, as a result of both dynamic and thermodynamic preference, most of the H2TPyP molecules are inclined to form H-type molecular associations suddenly via intermolecular π-π interactions without the formation of a H2TPyP/CTAB complex. In this sense, the role mainly played by CTAB is to help the oil phase to form spherical emulsions (we find that when an anionic surfactant, sodium *n*-dodecyl sulfate, with an appropriate concentration is employed, nanostructures predominately occupied by shuttle-like nanoarchitectures could also be formulated; figures not shown herein). With the volatilization of the chloroform solvent, the spherical emulsion shrivels in a manner similar to a deflated ball, resulting in a predominant formation of shuttle-like nanostructures. In the case of a CATB aqueous solution with a lower concentration, owing to the deficiency of surfactants, only a certain partial of the oil phases could form well-defined spherical emulsions, resulting in the formation of some shuttle-like nanostructures together with some irregular nanostructures.

## Conclusions

In summary, we have shown that when a surfactant aqueous solution with an appropriate concentration is selected, a free-base porphyrin, H2TPyP, could be assembled to predominately form unique yet well-defined shuttle-like nanoarchitectures via SASA in an oil/water medium. This might be the first report of porphyrin-based shuttle-like nanoarchitectures which could be facilely produced. By taking into account the molecular geometry of H2TPyP, the intermolecular non-covalent interactions, as well as the role of the surfactant, a possible explanation has been proposed. It gives deep insights into the self-assembly behavior of porphyrins in an oil/water system and provides important clues concerning the design of appropriate porphyrins when related subjects are addressed. Our investigation suggests that an oil/aqueous system might be an efficient medium for producing organic-based nanostructures with unique yet well-defined morphologies.

## Abbreviations

H2TPyP: 5, 10, 15, 20-tetra(4-pyridyl)-21*H*, 23*H*-porphine; CTAB: cetyltrimethylammonium bromide; SASA: surfactant-assisted self-assembly; ZnTPyP: zinc 5, 10, 15, 20-tetra(4-pyridyl)-21*H*, 23*H*-porphine; LRTEM: low-resolution transmission electron microscopy; HRTEM: high-resolution transmission electron microscopy; FFT: fast Fourier transformation; EDX: energy-dispersive X-ray spectroscopy; SEM: scanning electron microscopy.

## Competing interests

The authors declare that they have no competing interests.

## Authors' contributions

The authors of this paper contributed equally to this work.
